# A Short-Term High-Sugar Diet Induces Glucose Intolerance, Visceral Adipose Tissue Inflammation, and Exacerbates Experimental Allergic Asthma

**DOI:** 10.3390/nu18091475

**Published:** 2026-05-06

**Authors:** Mateus C. Casaro, Vanessa de Souza, Eduardo Mendes, Juliana Carvalho Pereira, Fernando A. Oliveira, Caroline M. Ferreira

**Affiliations:** 1 Department of Pharmaceutics Sciences, Institute of Environmental, Chemistry and Pharmaceutical Sciences, Universidade Federal de São Paulo, R. São Nicolau, 210, Diadema 09913-03, SP, Brazil; mateuscasaro@gmail.com (M.C.C.); vsouza@unifesp.br (V.d.S.); eduardo.mendes@unifesp.br (E.M.);; 2 Cellular and Molecular Neurobiology Laboratory (LaNeC), Center for Mathematics, Computing and Cognition (CMCC), Federal University of ABC—UFABC, São Bernardo do Campo 09606-045, SP, Brazil; oliveira.fernando@ufabc.edu.br

**Keywords:** adipokines, asthma, glucose intolerance, high-sugar diet, inflammation, visceral adipose tissue

## Abstract

Background: Diets composed of various components have been shown to influence inflammatory diseases such as asthma. While most studies have focused on fiber-rich diets to investigate their effects on the immune system and, consequently, on asthma, little is known about the impact of sugar-rich diets, particularly when such diets are consumed over short periods of time. Methods: To investigate the short-term effects of a sugar-rich diet on allergic airway inflammation, A/J mice were fed either a standard diet or a sugar-enriched diet and subsequently sensitized and challenged with ovalbumin or PBS. Airway inflammation was assessed by bronchoalveolar lavage (BAL) cell analysis, including eosinophil counts and cytokine levels (IL-4, TNF-α, IL-33), and by lung histology (H&E for inflammatory infiltrate and PAS for mucus). Serum IgE levels were also measured. In addition, glucose tolerance, visceral and subcutaneous adipose tissue mass, and inflammatory markers in visceral adipose tissue were evaluated. Results: Short-term consumption of a sugar-rich diet induced glucose intolerance and expansion of adipose tissue, particularly visceral fat, independent of ovalbumin sensitization. Gonadal adipose tissue analysis revealed a shift toward M1 macrophage polarization, characterized by elevated TNF-α, IL-6, and IL-1β, increased leptin levels, and reduced adiponectin. In OVA-sensitized mice, the sugar-rich diet significantly exacerbated eosinophil infiltration in BAL, increased IL-4, TNF-α, and IL-33, and enhanced PAS-positive mucus accumulation and inflammatory infiltrates in the lung. Moreover, total serum IgE was significantly higher in allergic mice fed the sugar-rich diet compared with allergic mice on the standard diet. Importantly, in non-sensitized mice fed the sugar-rich diet, no pulmonary inflammation was detected by BAL, demonstrating that HSD alone does not induce asthma but amplifies allergic responses when sensitization is present. Conclusions: Our findings demonstrate that short-term consumption of a sugar-rich diet is sufficient to exacerbate, but not initiate, allergic pulmonary inflammation. From a translational perspective, reducing dietary sugar intake may represent a valuable adjuvant strategy in the management of allergic asthma.

## 1. Introduction

The prevalence of non-communicable chronic diseases (NCDs), such as allergic asthma, has risen worldwide in recent decades, particularly in Western societies, driven by multifactorial causes including high-calorie diets [1,2,3]. Diet is recognized as an important factor contributing to the development and progression of NCDs. One of the most striking changes has been the dramatic increase in sugar and refined carbohydrate consumption [4,5,6]. According to the US Census Bureau, the average American consumes 22 pounds (approximately 10 kg) of candy annually [7]. In Brazil, a survey by Kantar WorldPanel reported that 99.7% of families consumed cookies in 2020 [8]. Similarly, in the United Kingdom, 65% of calories consumed by school-aged children came from white bread, biscuits, carbonated drinks, crisps, and chips [9,10,11,12,13,14]. Epidemiological studies have consistently linked the intake of soft drinks and other sugary beverages to inflammatory diseases [15,16,17].

Excess caloric intake is associated with chronic low-grade systemic inflammation and adipose tissue dysfunction [18,19,20]. High sugar consumption has been implicated in metabolic [21,22,23], gastrointestinal [21,24], and inflammatory [25,26] disorders. Asthma, a disease influenced by diet, gut microbiota, and metabolism, may also be affected by these dietary patterns. While high-fiber diets that promote short-chain fatty acid production are known to improve asthma outcomes [27,28], diets rich in sugar appear to have the opposite effect, promoting airway inflammation. For instance, soft drinks high in fructose have been associated with enhanced activation of the pulmonary immune system [29]. Excessive sugar intake has also been linked to increased risk of childhood asthma [28,30,31,32,33].

Visceral adipose tissue (VAT) plays a particularly important role in this context. Compared with subcutaneous adipose tissue, VAT shows greater infiltration of M1 pro-inflammatory macrophages and reduced adiponectin production, creating a pro-inflammatory environment that can exacerbate allergic airway responses [34,35]. Although the impact of long-term obesogenic diets on asthma is well established [36,37], there are no experimental studies directly evaluating the short-term effects of high-sugar diets (HSD) on allergic lung disease.

To address this gap, we investigated the effects of HSD on glucose tolerance, adipose tissue inflammation, and lung inflammation in an experimental model of asthma using A/J mice, which are highly susceptible to allergic airway inflammation. Mice were fed a sucrose-rich diet containing condensed milk, based on findings by Masi et al. [5], who showed that high-sucrose diets induce greater lipogenesis and a more pro-inflammatory profile than high-fat diets in C57BL/6 mice. The HSD was administered for 15 days prior to asthma induction to assess metabolic changes and continued throughout the experimental period to evaluate its impact on allergic airway inflammation. Condensed milk was chosen as a sucrose-rich source due to its standardized composition, high palatability, and widespread consumption in several countries, including Brazil, the United States, Vietnam, Russia, and Mexico, where it is commonly used in desserts and beverages. This enhances the translational relevance of our experimental model.

Our results demonstrate that HSD rapidly induces glucose intolerance and preferential visceral adipose expansion, independent of allergic sensitization. However, in the context of allergic asthma, HSD markedly exacerbates eosinophilic lung inflammation, Th2 cytokine production, and PAS-positive mucus accumulation due to goblet cell hyperplasia. These findings suggest that even short-term consumption of a high-sugar diet can worsen allergic asthma through combined metabolic and adipose tissue-mediated inflammatory pathways.

## 2. Materials and Methods

### 2.1. Animals

Female A/J mice (6–8 weeks old) were obtained from the animal facility of the Institute of Biomedical Sciences, University of São Paulo, Brazil. Mice were housed under specific pathogen-free conditions with controlled temperature (22 ± 2 °C), humidity (55 ± 10%), and a 12 h light/dark cycle. All mice were age-matched and initially fed a standard chow diet (Nuvilab CR-1, Quimtia, São Paulo, Brazil). At the end of the experimental protocol, mice were euthanized with a high dose of ketamine and xylazine administered intraperitoneally. After loss of reflexes and deep anesthesia, thoracic cavity opening was performed to ensure death. All protocols were approved by the Ethics Committee on Animal Research of the Federal University of São Paulo (Protocol 9742150818) and were conducted in accordance with the guidelines of the Brazilian Council for the Control of Animal Experimentation (CONCEA) and the ARRIVE guidelines.

### 2.2. Dietary Intervention

The mice were fed a control diet (CD), which consisted of the standard Nuvilab CR-1 diet (manufacturer’s technical sheet), composed of 76% carbohydrates, mainly complex polysaccharides and fiber; 15% protein from soybean meal and wheat bran; 9% fat primarily from soybean oil rich in linoleic acid. The high-sugar diet (HSD) group received the CD plus a separate bowl of condensed milk (Italac, São Paulo, Brazil; nutritional label), composed of 68% carbohydrates (predominantly sucrose and lactose, with negligible fiber), 23% fat (mainly saturated milk fat with smaller amounts of oleic and linoleic acids), and 9% protein (casein and whey). Both diets, condensed milk, and water were provided ad libitum. Dietary intervention with condensed milk started on day 15, and mice were maintained on this diet for fifteen consecutive days before the first OVA sensitization (day 0 of the asthma induction protocol). Diets were maintained until the end of the study (day 21 after sensitization, corresponding to day 37 of the overall protocol). Food consumption was monitored daily by weighing the chow and condensed milk provided and subtracting the remaining amount at the next measurement. Caloric intake was calculated using the manufacturer’s nutritional information (Nuvilab CR-1 technical sheet and Italac condensed milk label).

### 2.3. Experimental Groups

Mice were randomly assigned to four experimental groups (*n* = 6–9 per group): (1) Saline-CD: Saline-sensitized and challenged; fed standard chow diet, (2) Saline-HSD: Saline-sensitized and challenged; fed standard chow and condensed milk ad libitum, (3) OVA-CD: OVA-sensitized and challenged; fed standard chow diet, (4) OVA-HSD: OVA-sensitized and challenged; fed standard chow and condensed milk ad libitum. Metabolic parameters (glucose tolerance, body weight, adipose tissue mass, serum lipids) were assessed in all four groups. Lung inflammation parameters (bronchoalveolar lavage cell counts, cytokines, histology) were assessed in OVA-sensitized groups (OVA-CD and OVA-HSD), as Control groups (Control-CD and Control-HSD) showed no significant airway inflammation (100% mononuclear cells in BALF, no eosinophils, no PAS-positive mucus accumulation).

### 2.4. Induction of Allergic Lung Inflammation

For the HSD group, condensed milk was introduced on day 15 of the experimental protocol, and mice were maintained on this diet for fifteen days before the first OVA sensitization (day 0 of the asthma induction protocol). Mice were sensitized via intraperitoneal (i.p.) injection with 50 μg of OVA grade V (Sigma Chemical Co., St. Louis, MO, USA) dissolved in 200 μL of sterile phosphate-buffered saline (PBS) and 1.6 mg of aluminum hydroxide adjuvant (Imject Alum, Prod# 77161, Thermo Scientific, Rockford, IL, USA) on days 15 and 22. Mice were challenged with 50 μg of OVA in 50 μL of sterile PBS via intratracheal (i.t.) administration on days 29 and 36. Control mice received intraperitoneal and intratracheal administrations of sterile saline instead of OVA, following the same schedule and procedures as the experimental groups [38].

### 2.5. Metabolic Parameters

#### 2.5.1. Oral Glucose Tolerance Test (OGTT)

Mice were fasted for 6 h during the light phase (08:00–14:00) of the light/dark cycle, when food intake is naturally reduced, and body weight was recorded. No OGTT was performed at baseline (day 0); the test was conducted only on day 14 (one day before the first OVA sensitization) to assess early metabolic changes induced by HSD. The tip of each mouse’s tail was nicked using sterile surgical scissors, and a blood drop was collected directly onto a glucose meter test strip (Accu-Chek Active^®^, Roche Diagnostics GmbH, Mannheim, Germany) to measure baseline glucose levels (time 0). A 25% glucose solution was then administered via oral gavage at a dose of 8 μL per gram of body weight. Blood glucose levels were subsequently measured at 15, 30, 45, 60, and 75 min after glucose administration. A glucose concentration–time curve was plotted, and the area under the curve (AUC) was calculated for each animal using the trapezoidal method [39].

#### 2.5.2. Serum Biochemical Analyses

At euthanasia (day 37), blood samples were collected via cardiac puncture after a 6 h fast. Serum was separated by centrifugation (3000 rpm, 15 min, 4 °C) and stored at −80 °C. Fasted serum glucose, total cholesterol, LDL-cholesterol, and HDL-cholesterol were measured using colorimetric assay kits (Labtest Diagnóstica, Lagoa Santa, Brazil) according to the manufacturer’s instructions.

### 2.6. Adipose Tissue Collection and Processing

At euthanasia (day 37), four distinct white adipose tissue depots were carefully dissected and weighed: gonadal (periovarian in females), retroperitoneal, inguinal subcutaneous, and interscapular brown adipose tissue. Total visceral adipose tissue (VAT) mass was calculated as the sum of gonadal and retroperitoneal depot weights. The VAT/subcutaneous adipose tissue (SAT) ratio was determined. Gonadal adipose tissue was selected for detailed molecular analyses due to its high metabolic activity, large depot size, and established role in metabolic inflammation [24,40]. Tissue samples were either snap-frozen in liquid nitrogen and stored at −80 °C for gene expression analyses.

### 2.7. Gene Expression Analysis

The gene expression of M1 macrophage markers (iNOS, CD11c), M2 macrophage markers (Arginase-1, CD206), pro-inflammatory cytokines (TNF-α, IL-6, IL-1β), regulatory cytokine (IL-10 and IL-5), and adipokines (Leptin, Adiponectin) in gonadal adipose tissue was evaluated by quantitative real-time PCR (qRT-PCR). Briefly, total RNA was extracted from adipose tissue using TRIzol reagent (Invitrogen, Carlsbad, CA, USA) according to the manufacturer’s protocol. RNA concentration and purity were assessed by spectrophotometry (NanoDrop, Thermo Fisher Scientific, Waltham, MA, USA). One microgram of total RNA was reverse transcribed into cDNA using the High,-Capacity cDNA Reverse Transcription Kit (Applied Biosystems, Foster City, CA, USA). qRT-PCR reactions were performed in a Rotor Gene Q system (Qiagen, Hilden, Germany) using SYBR Green as the fluorescent dye (Platinum^®^ SYBR^®^ Green qPCR SuperMix-UDG, Invitrogen). Each reaction contained 50 ng of cDNA template and primers at a final concentration of 0.5 µM. Beta-2-microglobulin (B2M) was used as the housekeeping gene for normalization. Primer sequences and concentrations are described in Supplementary Table S1. The relative gene expression was calculated using the 2^−ΔΔCT^ method [41]. Results are expressed as fold change relative to the OVA-CD group. Primer sequences are listed in Supplementary Table S1.

### 2.8. Bronchoalveolar Lavage Fluid (BALF) Collection and Analysis

Twenty-four hours after the last OVA challenge, mice were euthanized, and the trachea was cannulated with a polyethylene catheter. Bronchoalveolar lavage was performed by instilling and recovering 1 mL of sterile PBS (3 × 0.3 mL) into the lungs. BALF samples were centrifuged at 1200 rpm for 5 min at 4 °C. Cell pellets were resuspended in PBS, and total cell counts were performed using a hemocytometer. Differential cell counts (eosinophils, neutrophils, and mononuclear cells) were determined on cytospin preparations stained with Diff-Quik (Laborclin, Pinhais, Brazil) by counting at least 300 cells per slide under light microscopy. BALF supernatants were stored at −80 °C for cytokine and immunoglobulin measurements [38].

### 2.9. Cytokines and IgE Measurements

Cytokine concentrations in BALF were measured using enzyme-linked immunosorbent assay (ELISA) kits for IL-4, IL-5, IL-10 (BD Biosciences, San Diego, CA, USA), and IL-13, IL-33, TNF-α (R&D Systems, Minneapolis, MN, USA) according to the manufacturers’ instructions. Serum total IgE and OVA-specific IgE levels were measured using ELISA kits from BD Biosciences and Cayman Chemical (Ann Arbor, MI, USA), respectively. All determinations were performed in duplicate [6,34].

### 2.10. Histological Analysis of the Lung

The left lung lobe was fixed in 10% buffered formalin solution immediately after bronchoalveolar lavage. Tissues were processed, embedded in paraffin, sectioned at 5 μm thickness, and stained with hematoxylin and eosin (H&E) to evaluate leukocyte infiltration, or with periodic acid-Schiff (PAS) reagent to assess goblet cell hyperplasia and PAS-positive mucus accumulation. For peribronchial and perivascular inflammation scoring, all bronchi and vessels in each histological section were analyzed blindly (without knowledge of group assignment) by at least two independent observers. Inflammation was scored on a scale of 0–4 based on the density and extent of inflammatory cell infiltration: 0 = no inflammation; 1 = mild; 2 = moderate; 3 = marked; 4 = severe [38]. For mucus analysis, PAS-stained sections were scored based on the percentage of PAS-positive epithelial cells in each bronchus: 0 = no PAS-positive cells; 1 = up to 25% of the bronchial circumference; 2 = 26–50%; 3 = 51–75%; 4 ≥ 75% [38]. The proportion of bronchi with mucus (PAS-positive) was also calculated.

### 2.11. Statistical Analysis

Descriptive statistics were performed using GraphPad Prism 7 (GraphPad Software, San Diego, CA, USA). Data are presented as means ± standard error of the mean (SEM). Comparisons between two groups were performed using Student’s *t*-test (unpaired, two-tailed) after confirming normal distribution (Shapiro–Wilk test) and equal variances (F-test). For comparisons involving multiple time points (e.g., glucose tolerance test, body weight progression), two-way ANOVA followed by Bonferroni post hoc test was used. A *p*-value ≤ 0.05 was considered statistically significant. Statistical significance is indicated as follows: * *p* < 0.05, ** *p* < 0.01, *** *p* < 0.001, **** *p* < 0.0001.

## 3. Results

### 3.1. HSD Rapidly Induces Glucose Intolerance Before Asthma Induction

**Figure 1:**
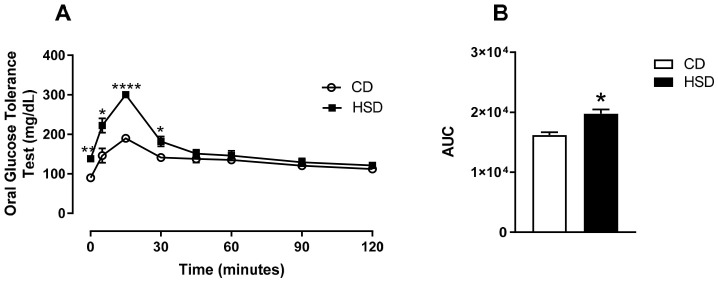
Metabolic profile in A/J mice fed a control diet (CD) or high-sugar diet (HSD). (**A**) Blood glucose levels during the oral glucose tolerance test (OGTT) performed on day 14 (one day before the first OVA sensitization). Mice were fasted for 6 h, and glucose was administered via oral gavage (2 g/kg body weight). Blood glucose was measured at 0, 15, 30, 45, 60, and 75 min. (**B**) Area under the curve (AUC) of blood glucose during the OGTT, calculated using the trapezoidal method. For panel A, two-way ANOVA followed by Bonferroni post hoc test was used. For graph B, Student’s *t*-test was used to compare CD and HSD groups. * *p* < 0.05; ** *p* < 0.01; **** *p* < 0.0001.

To evaluate the early metabolic effects of HSD independent of allergic inflammation, we performed an oral glucose tolerance test (OGTT) on day 14, one day before the first OVA sensitization. Mice fed HSD for 14 days exhibited significantly impaired glucose tolerance compared with control diet (CD)-fed mice, as demonstrated by elevated blood glucose levels at multiple time points during the OGTT (Figure 1A). The area under the curve (AUC) for blood glucose was 22% higher in HSD-fed mice compared with CD-fed mice (Figure 1B, *p* < 0.05), indicating early glucose intolerance induced by short-term high-sugar consumption. Energy intake was monitored daily by weighing food and condensed milk, and calculating caloric intake based on manufacturer’s nutritional information. Mean daily caloric intake was 10.05 ± 0.87 kcal in Saline-CD, 13.23 ± 2.02 kcal in Saline-HSD, 9.43 ± 1.49 kcal in OVA-CD, and 12.16 ± 2.35 kcal in OVA-HSD. Thus, energy intake was not similar among the groups, with significantly higher values in the high-sugar diet groups. Statistical analysis confirmed that energy intake was significantly higher in the high-sugar diet groups compared with control diet groups (*p* < 0.05).

### 3.2. HSD Promotes Body Weight Gain and Preferential Visceral Adipose Tissue Expansion

After the initial 15-day dietary intervention, experimental asthma was induced with OVA, and the diets were maintained until the end of the study (day 37). Consistent with the higher caloric intake observed in HSD-fed mice, condensed milk consumption increased while chow intake decreased. Food intake was similar between OVA-sensitized and non-sensitized mice within each diet group (*p* > 0.05), indicating that allergic sensitization did not affect feeding behavior (Table 1).

At baseline, OVA-sensitized mice presented lower initial body weight compared with non-sensitized controls. By day 37, HSD feeding led to a consistent increase in body weight gain in both saline and OVA groups. Saline-HSD mice gained about 6.5 g, while OVA-HSD mice gained around 3.9 g, significantly higher than their respective CD controls. In contrast, OVA-CD mice showed minimal weight gain (0.5 g) (Table 1).

Analysis of adipose tissue depots revealed that HSD significantly increased gonadal, retroperitoneal, subcutaneous, and brown adipose tissue in non-sensitized mice. OVA-CD mice exhibited markedly reduced depot weights, whereas OVA-HSD mice showed partial recovery, with values intermediate between OVA-CD and Saline-HSD. Total visceral adipose tissue (VAT, calculated as gonadal + retroperitoneal) was increased in both HSD groups, with OVA-HSD mice showing a 2.9-fold increase compared with OVA-CD. The VAT/SAT ratio, however, did not differ significantly among groups, indicating that sugar intake promoted overall adipose expansion rather than selective visceral accumulation (Table 1).

### 3.3. HSD Alters Serum Lipid Profile Without Affecting Fasted Glucose at Day 37

At the end of the experimental period (day 37), fasting serum glucose was significantly increased in Saline-HSD compared with Saline-CD, while OVA-CD mice showed lower values compared with Saline-HSD (Table 1). OVA-HSD animals displayed intermediate levels without statistical difference (Table 1). Serum lipid profiles were markedly altered by HSD: total cholesterol increased by 77%, LDL-cholesterol by 62%, and HDL-cholesterol by 31% in Saline-HSD compared with Saline-CD. Similar alterations were observed in OVA-sensitized mice, although OVA-CD animals exhibited reduced cholesterol and LDL levels relative to controls. OVA-HSD mice exhibited glucose and triglyceride levels that were neither significantly elevated nor reduced compared with the other groups (Table 1).

### 3.4. HSD Induces Pro-Inflammatory Phenotype in Gonadal Adipose Tissue

Given the marked expansion of visceral adipose tissue, we next evaluated the inflammatory profile of gonadal adipose tissue (gAT), the largest and most metabolically active visceral depot. Gene expression analysis revealed that HSD promoted a shift toward a pro-inflammatory M1 macrophage phenotype in gAT. The mRNA expression of M2 macrophage markers (Arginase-1 and CD206) was significantly reduced (Table 2, *p* < 0.01 and *p* < 0.0001, respectively), while M1 macrophage markers (iNOS and CD11c) were markedly increased (Table 2, *p* < 0.05 and *p* < 0.0001, respectively) in the OVA-HSD group compared with the OVA-CD group.

Pro-inflammatory cytokines TNF-α, IL-6, and IL-1β were significantly elevated in gAT of OVA-HSD mice (Table 2, all *p* < 0.0001), whereas regulatory cytokine IL-10 and IL-5 were reduced (Table 2, *p* < 0.0001). Furthermore, leptin mRNA expression increased by 2.6-fold, while adiponectin mRNA expression decreased by 2.9-fold in the OVA-HSD group compared with the OVA-CD group (Table 2, both *p* < 0.0001). These findings indicate that HSD induces adipose tissue inflammation characterized by M1 macrophage polarization, elevated pro-inflammatory cytokines, and adipokine dysregulation.

### 3.5. HSD Exacerbates Eosinophilic Airway Inflammation in OVA-Sensitized Mice

To evaluate the impact of HSD on allergic lung inflammation, we analyzed bronchoalveolar lavage fluid (BALF) from all experimental groups. As expected, sensitization and challenge with OVA induced leukocyte infiltration with marked eosinophilia in the airways of A/J mice [42]. Non-OVA-sensitized mice (Control-CD and Control-HSD groups) exhibited 100% mononuclear cells in BALF with no eosinophils or neutrophils, and HSD alone did not alter this cellular profile, indicating that HSD does not initiate allergic airway inflammation in the absence of allergen sensitization.

**Figure 2:**
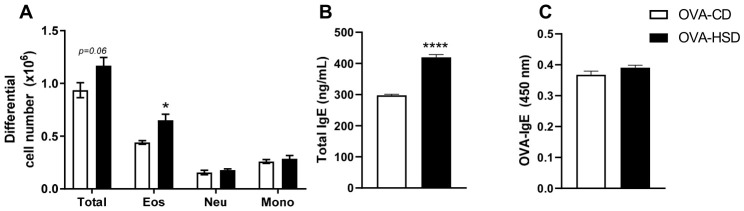
HSD exacerbates eosinophilic airway inflammation in OVA-sensitized mice. (**A**) Total and differential cell counts in BALF from OVA-CD and OVA-HSD groups, determined by hemocytometer and Diff-Quik-stained cytospin preparations. (**B**) Serum total IgE levels measured by ELISA at day 37. (**C**) Serum OVA-specific IgE levels measured by ELISA at day 37. Data are presented as mean ± SEM (*n* = 6–9 per group). * *p* < 0.05, **** *p* < 0.0001 compared with OVA-CD (Student’s *t*-test).

In contrast, OVA-sensitized mice fed HSD (OVA-HSD) exhibited increased total cell infiltration in the airways compared with OVA-CD mice (*p*= 0.06), driven primarily by enhanced eosinophil recruitment (Figure 2A, *p* < 0.05). Neutrophil counts were also elevated, though to a lesser extent, while mononuclear cell numbers remained similar between OVA-CD and OVA-HSD groups (Figure 2A).

Serum total IgE levels were significantly higher in OVA-HSD mice compared with OVA-CD mice (Figure 2B, *p* < 0.0001), indicating enhanced systemic allergic sensitization. However, OVA-specific IgE levels were not significantly different between the two groups (Figure 2C), suggesting that HSD exacerbates allergic inflammation through mechanisms beyond antigen-specific antibody production.

### 3.6. HSD Intensifies Peribronchial and Perivascular Inflammation and Goblet Cell Hyperpasia (PAS Staining) in Bronchi

**Figure 3:**
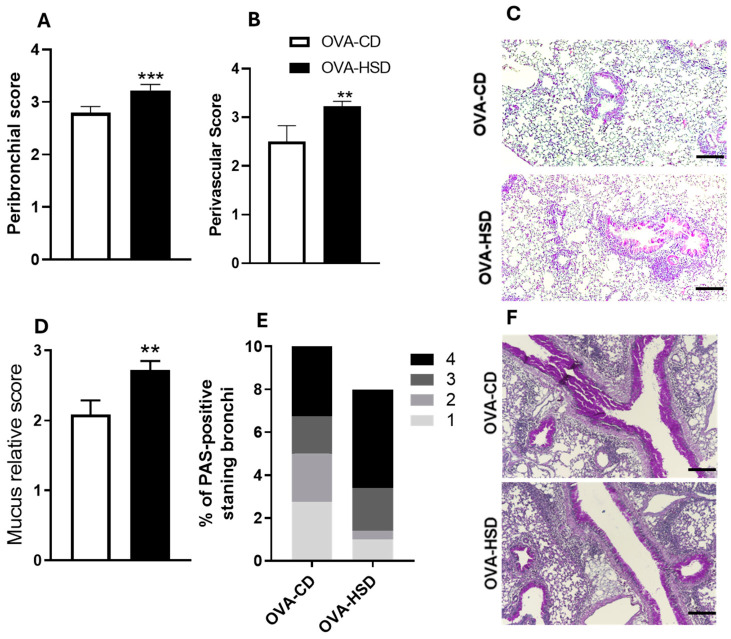
HSD intensifies peribronchial and perivascular inflammation and PAS-positive mucus accumulation in OVA-sensitized mice. (**A**) Peribronchial inflammation score assessed on hematoxylin and eosin (H&E)-stained lung sections. All bronchi in each section were scored blindly on a scale of 0–4 based on inflammatory cell infiltration density: 0 = no inflammation; 1 = mild; 2 = moderate; 3 = marked; 4 = severe. (**B**) Perivascular inflammation score assessed on H&E-stained sections using the same scoring system. (**C**) Representative H&E-stained lung sections showing dense inflammatory infiltrates surrounding bronchi and blood vessels in OVA-HSD mice compared with OVA-CD mice. Note the increased peribronchial and perivascular cellularity, thickened bronchial walls, and enhanced inflammatory cell accumulation in OVA-HSD group. Scale bar: 50 μm; original magnification ×200. (**D**) Goblet cell hyperplasia score assessed on PAS-stained lung sections. Each bronchus was scored based on the percentage of PAS-positive epithelial cells: 0 = no PAS staining; 1 = up to 25% of bronchial circumference; 2 = 26–50%; 3 = 51–75%; 4 ≥ 75%. (**E**) Proportion of PAS-positive bronchi (bronchi with mucus) expressed as percentage of total bronchi analyzed. (**F**) Representative PAS-stained lung sections showing abundant PAS-positive mucus (magenta/purple staining) in bronchial epithelial cells and airway lumens of OVA-HSD mice compared with OVA-CD mice, indicating goblet cell hyperplasia. Scale bar: 50 μm; original magnification ×200. Data are presented as mean ± SEM (*n* = 6–9 per group), representative of at least five animals per group, ** *p* < 0.01, and *** *p* < 0.001 compared with the OVA-CD group (Student’s *t*-test).

OVA-HSD mice showed significantly higher peribronchial inflammation scores (Figure 3A, *p* < 0.001) and perivascular inflammation scores (Figure 3B, *p* < 0.01) compared with OVA-CD mice. Representative H&E-stained lung sections revealed increased inflammatory infiltrates surrounding bronchi and blood vessels in OVA-HSD mice (Figure 3C).

In addition to inflammation, goblet cell hyperplasia and PAS-positive mucus accumulation, important features of asthma, were also evaluated. PAS staining demonstrated that OVA-HSD mice exhibited enhanced goblet cell hyperplasia in the bronchial epithelium compared with OVA-CD mice (Figure 3D, *p* < 0.01). Figure 3E showed a higher percentage of high scores for PAS. This finding indicates enhanced goblet cell activity in the OVA-HSD group. Histological findings from H&E and PAS staining further support the quantitative data presented (Figure 3E,F).

### 3.7. HSD Elevates Th2 and Pro-Inflammatory Cytokines in BALF

**Figure 4:**
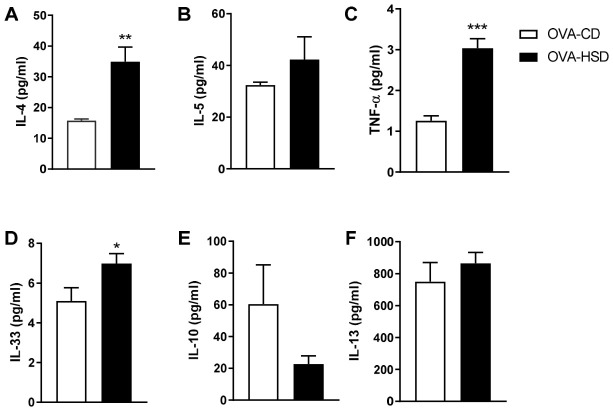
HSD elevates Th2 and pro-inflammatory cytokines in bronchoalveolar lavage fluid (BALF) of OVA-sensitized mice. Cytokine concentrations in BALF were measured by ELISA 24 h after the last OVA challenge (day 37). (**A**) IL-4; (**B**) IL-5; (**C**) TNF-α; (**D**) IL-33; (**E**) IL-10; (**F**) IL-13. Data are expressed as mean ± SEM (*n* = 6–9 per group). * *p* < 0.05, ** *p* < 0.01, and *** *p* < 0.001 compared with the OVA-CD group (Student’s *t*-test).

To further characterize the exacerbation of allergic airway inflammation by HSD, we measured cytokine levels in BALF. HSD significantly increased the levels of key Th2 cytokines and pro-inflammatory mediators. IL-4, a critical Th2 cytokine driving IgE production and eosinophil recruitment, was elevated by 2.5-fold in OVA-HSD mice compared with OVA-CD mice (Figure 4A, *p* < 0.01). IL-33, an alarmin that amplifies Th2 responses, was increased by 3.2-fold (Figure 4D, *p* < 0.05). TNF-α, a pro-inflammatory cytokine implicated in asthma severity, was elevated by 2.8-fold (Figure 4C, *p* < 0.001).

In contrast, IL-5 (Figure 4B), IL-10 (Figure 4E), and IL-13 (Figure 4F) levels in BALF were not significantly different between OVA-CD and OVA-HSD groups, suggesting that HSD selectively enhances specific inflammatory pathways (IL-4, IL-33, TNF-α) rather than globally amplifying all Th2 cytokines.

## 4. Discussion

**Figure 5:**
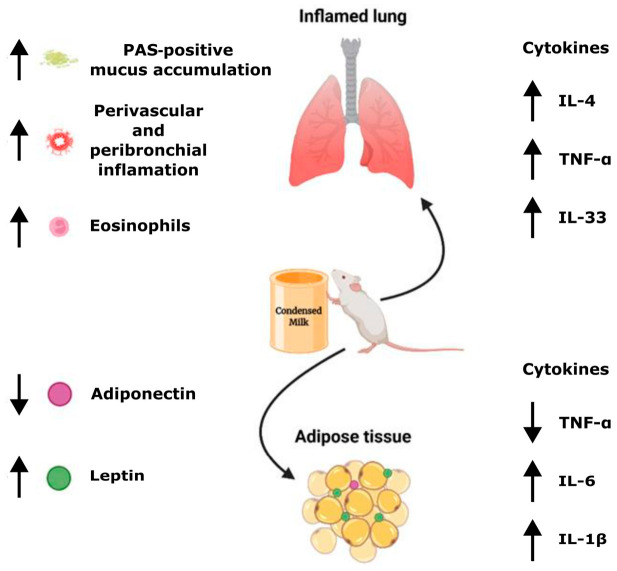
Schematic representation of the mechanisms by which short-term high-sugar diet (HSD) exacerbates allergic asthma. HSD consumption leads to expansion of visceral adipose tissue (VAT) that is accompanied by elevated pro-inflammatory cytokines (TNF-α, IL-6, IL-1β), increased leptin secretion, and decreased adiponectin levels in adipose tissue. These adipose-derived inflammatory mediators enter the systemic circulation and contribute to a pro-inflammatory systemic environment. In the context of allergic sensitization (OVA), this systemic inflammation exacerbates airway inflammation, characterized by increased eosinophil recruitment, elevated Th2 cytokines (IL-4, IL-33), enhanced TNF-α production, intensified peribronchial and perivascular inflammation, and PAS-positive mucus accumulation by goblet cells. Notably, lung inflammation does not arise only from systemic cytokines but requires pre-existing allergenic sensitization, as non-sensitized mice did not develop airway inflammation despite systemic inflammation. Upward arrows indicate increase, downward arrows indicate reduction. Original illustration created by the authors using BioRender.com.

There are several studies that provide obesogenic diets to mice for extended periods (8–12 weeks) to induce obesity or diabetes and investigate the consequences on inflammatory lung diseases [36,37,40]; however, studies directly associating short-term high-sugar diets with allergic conditions, such as asthma, are scarce. In our study, the goal was not to induce obesity but to investigate the short-term effects of HSD on metabolic dysregulation and its impact on immunological changes in asthma. Remarkably, only 15 days of HSD were sufficient to induce glucose intolerance compared with control diet-fed animals. By the end of the experimental protocol (37 days), HSD-fed mice exhibited significant body weight gain, preferential visceral adipose tissue expansion, and marked alterations in metabolic and inflammatory parameters (Figure 5).

The diet used in this study was based on the findings of Masi et al., who provided C57BL/6 mice with three obesogenic diets for 8 weeks: a high-fat diet, a high-sucrose diet with condensed milk, and a combination of both [5]. All diets induced weight gain and insulin resistance; however, the high-sucrose diet promoted greater lipogenesis and a more pro-inflammatory profile compared with the high-fat diet alone. Similarly, in BALB/c mice, condensed milk and refined sugar rapidly expanded adipose tissue and increased leukocytes, pro-inflammatory cytokines, and enzymes involved in lipogenesis [24]. Ferreira et al. demonstrated that a high-sucrose diet mainly increases fat synthesis and adipogenesis through enhanced lipogenesis, whereas a high-fat diet decreases lipolytic activity without activating lipogenic pathways [24].

Considering that sugar can drive metabolic and immunological changes [41] and that insulin-resistant patients are more prone to asthma symptoms [30,31,43], we evaluated the impact of short-term HSD on metabolic alterations and their association with experimental asthma. Asthma was induced using OVA, and we observed exacerbated eosinophilic inflammation in HSD-fed mice, accompanied by elevated IL-4, IL-33, and TNF-α in the bronchoalveolar lavage fluid. Goblet cell hyperplasia and PAS-positive mucus accumulation were also markedly increased. Although OVA-specific IgE did not differ between groups, total IgE was significantly elevated in sugar-fed mice, suggesting that dietary sugar may enhance systemic allergic activation beyond antigen-specific responses. This is the first experimental study linking short-term HSD with asthma exacerbation and adipose tissue changes.

Importantly, the metabolic effects of HSD were independent of allergic sensitization status. Both OVA-sensitized and non-sensitized mice fed HSD showed similar increases in body weight, adipose tissue expansion, and alterations in serum lipid profiles (total cholesterol, LDL-cholesterol, and HDL-cholesterol). However, allergic sensitization itself influenced some parameters: OVA-CD animals gained minimal weight, exhibited reduced adipose depots, and presented lower cholesterol and LDL levels compared with controls. When exposed to HSD, OVA-sensitized mice partially recovered adipose tissue and displayed fasting glucose values that fell between those of Saline-HSD and OVA-CD groups. At day 37, fasting serum glucose was significantly increased in Saline-HSD compared with Saline-CD, while OVA-CD mice showed lower values, and OVA-HSD animals exhibited intermediate levels without statistical difference. This pattern reinforces that allergic sensitization modulates glucose metabolism, attenuating the impact of HSD on fasting glycemia. This indicates that while the metabolic dysregulation induced by HSD is primarily a direct consequence of dietary sugar intake, the allergic inflammatory process exerts a modulatory effect, attenuating lipid and adipose accumulation under control diet and shaping the metabolic response to HSD.

Notably, non-sensitized mice (Control-CD and Control-HSD) exhibited 100% mononuclear cells in bronchoalveolar lavage fluid with no eosinophilic inflammation or PAS-positive mucus accumulation, confirming that HSD alone does not induce allergic airway inflammation in the absence of allergen sensitization. These findings support the concept that HSD acts as an exacerbating factor rather than an initiating trigger for allergic asthma. The metabolic and adipose tissue changes induced by HSD create a systemic pro-inflammatory environment that amplifies allergic responses only when allergen sensitization is present.

Our study demonstrated preferential expansion of visceral adipose tissue depots (gonadal and retroperitoneal). This visceral fat accumulation is clinically relevant, as visceral adiposity is more strongly associated with metabolic dysfunction, systemic inflammation, and respiratory disease than subcutaneous fat [44,45]. Visceral adipose tissue plays a critical role in asthma pathophysiology due to its heightened metabolic activity and inflammatory potential compared with subcutaneous fat [29]. Visceral fat accumulation is strongly associated with systemic inflammation, as it releases larger quantities of pro-inflammatory adipokines and free fatty acids directly into the portal circulation, affecting multiple organ systems, including the lungs [27].

In obesity, expansion of visceral abdominal adipose tissue restricts diaphragmatic contraction, thereby impairing pulmonary function. This mechanical limitation may compromise the physical defense mechanisms of the lungs, as immune cells depend on a healthy pulmonary environment with adequate airflow, oxygenation, and airway clearance. [28,33]. Furthermore, visceral adipose tissue exhibits a pro-inflammatory milieu, characterized by increased infiltration of M1 macrophages and reduced adiponectin production [34,35]. The gonadal adipose depot, which we selected for detailed molecular analysis, exhibited marked M1 macrophage polarization, elevated pro-inflammatory cytokines (TNF-α, IL-6, IL-1β), increased leptin, and decreased adiponectin expression. These changes in visceral adipose tissue likely contributed to the systemic inflammatory milieu that exacerbated allergic airway inflammation.

In our study, the consumption of condensed milk promoted the expansion of visceral adipose tissue, altering energy-regulating hormones, with increased leptin and decreased adiponectin levels. Adiponectin, usually anti-inflammatory, was decreased, whereas leptin was increased in OVA-HSD-fed mice. Leptin also influences immunity by promoting the production of TNF-α and IL-6, creating a bidirectional loop with pro-inflammatory cytokines such as TNF-α and IL-1 [46,47,48]. Leptin receptors in the lung can enhance survival, proliferation, and cytokine production in T lymphocytes [49,50]. Consistently, levels of TNF-α, IL-6, and IL-1β were higher in gonadal adipose tissue, and TNF-α was increased in the lungs of OVA-HSD-fed mice. The expansion of visceral adipose tissue observed in our HSD-fed mice likely contributed to the systemic inflammatory state, as this depot is particularly prone to macrophage infiltration and pro-inflammatory cytokine secretion, creating a link between metabolic dysfunction and pulmonary inflammation. Increased leptin correlates with body fat in obese asthma patients [51], and elevated leptin levels have been associated with asthma severity and reduced lung function [52]. Moreover, HSD promoted an increase in M1 adipose tissue macrophages (ATMs), which accumulate with increasing body weight, exhibit a “classically activated” inflammatory phenotype, and correlate with insulin resistance. Corroborating our findings, it has already been described that M1 adipose tissue macrophages secrete TNF-α and IL-6, as well as reactive oxygen species through iNOS activation, thereby impairing insulin signaling in adipocytes [27,53]. On the other hand, healthy adipose tissue from animals fed a balanced diet contains predominantly M2 macrophages, characterized by low production of inflammatory cytokines [54]. In adipose tissue, IL-5 and IL-10 play key roles in maintaining homeostasis. IL-5 sustains eosinophil populations that produce IL-4, promoting alternatively activated macrophages (M2) and supporting metabolic balance [54,55,56]. IL-10, in turn, is a well-established anti-inflammatory cytokine that suppresses pro-inflammatory responses. Together, they underscore the dual role of adipose tissue as both a source of inflammation and a site of immune regulation. It is also noteworthy that adipose tissue expansion has been shown to be associated with a reduction in regulatory T cells and, consequently, decreased production of the anti-inflammatory cytokine IL-10 [34,57], as observed in our study. We also highlight that female mice were deliberately chosen for this study. Asthma is more prevalent and often more severe in women, with symptoms frequently worsening in the premenstrual period [58]. This same phase is also associated with stronger cravings for sweet foods, which may increase susceptibility to the adverse effects of high-sugar diets [59,60]. Moreover, female animals have historically been underrepresented in biomedical research. Including them here helps to address this gap and makes our model more relevant to the human condition. Given the translational importance of this study, it is important to emphasize that the 37-day intervention period is relatively short compared with chronic dietary patterns in humans. It is important to acknowledge the limitations of this study, including the reliance on a murine model, the relatively short duration of dietary exposure, the fact that other systemic markers such as cytokines were not assessed, and the focus on an OVA-induced allergic asthma phenotype, which may not fully capture the heterogeneity of human asthma. Our work emphasizes the need for integrated approaches addressing both metabolic and immunological aspects of asthma, potentially opening new avenues for personalized therapeutic interventions targeting the adipose-lung axis.

## 5. Conclusions

In conclusion, our findings demonstrate that short-term consumption of a high-sucrose diet rapidly induces metabolic dysregulation, including glucose intolerance and dyslipidemia, and preferentially expands visceral adipose tissue, independent of allergic sensitization status. However, in the context of allergic asthma, HSD markedly exacerbates airway inflammation through adipose tissue-mediated mechanisms. The observed M1 macrophage polarization, adipokine dysregulation (increased leptin, decreased adiponectin), and elevated pro-inflammatory cytokines in visceral adipose tissue create a systemic pro-inflammatory environment that intensifies eosinophilic lung inflammation, Th2 cytokine production (IL-4, IL-33), TNF-α secretion, and PAS-positive mucus accumulation due to goblet cell hyperplasia. Importantly, HSD alone does not initiate allergic airway inflammation but acts as a powerful exacerbating factor in established asthma.

## Figures and Tables

**Table 1:** Body weight, serum metabolites, and adipose tissue depot weights in female A/J mice.

	Saline-CD	Saline-HSD	OVA-CD	OVA-HSD
Initial body weight (g)	20.95 ± 0.53	20.96 ± 1.4	18.53 ± 0.6	19 ± 0.46
Body weight gain (g)	1.57 ± 0.33	6.5 ± 0.6 ^a^	0.48 ± 0.23 ^b^	3.9 ± 0.4 ^a,b,c^
Total cholesterol (mg/dL)	104.9 ± 2.57	240.3 ± 13.98 ^a^	55.98 ± 13.53 ^a,b^	129.1 ± 4.0 ^b,c^
LDL-cholesterol (mg/dL)	80.22 ± 2.45	202.9 ± 11.48 ^a^	43.35 ± 12.98 ^b^	111.8 ±4.42 ^b,c^
HDL-cholesterol (mg/dL)	13.8 ± 0.99	32.49 ± 3.17 ^a^	7.08 ± 0.48 ^b^	9.23 ± 0.06 ^b^
Fasted serum glucose (mg/dL)	134 ± 6.32	251 ± 20.95 ^a^	158 ± 12.74 ^b^	186 ± 26.6
Triacylgycerol (mg/dL)	54.02 ± 4.18	24.68 ± 2.15 ^a^	27.69 ± 5.21 ^a^	40.14 ± 4.14 ^b^
Gonadal (mg)	0.48 ± 0.15	0.92 ± 0.27 ^a^	0.15 ± 0,09 ^a,b^	0.51 ± 0.11 ^b,c^
Retroperitoneal (mg)	0.21 ± 0.07	0.38 ± 0.09 ^a^	0.11 ± 0,02 ^b^	0.25 ± 0.09 ^b,c^
Subcutaneous (mg)	0.34 ± 0.11	0.53 ± 0.06	0.14 ± 0.06 ^b^	0.41 ± 0.15 ^c^
Brown adipose (mg)	0.11 ± 0.01	0.19 ± 0.06 ^a^	0.08 ± 0,01 ^b^	0.12 ± 0.02 ^c^
Total VAT (mg)	0.69 ± 0.22	1.31 ± 0.34 ^a^	0.26 ± 0.93 ^a,b^	0.76 ± 0.19 ^b,c^
VAT/SAT ratio	2.04 ± 0.08	2.49 ± 0.48	2.18 ± 0.87	1.99 ± 0.64

Initial body weight (day 0), body weight gain, serum metabolite levels (total cholesterol, LDL-cholesterol, HDL-cholesterol, triacylglycerol, and fasted serum glucose), and individual adipose tissue depot weights in female A/J mice at the end of the experimental period (day 37). Fasted serum glucose levels were measured after a 6 h fast. Adipose tissue depots were dissected and weighed at euthanasia (day 37). Total visceral adipose tissue (VAT) was calculated as the sum of gonadal and retroperitoneal depot weights. The VAT/SAT ratio was calculated as total VAT divided by subcutaneous adipose tissue (SAT) weight. Data are expressed as mean ± SEM (*n* = 4–5 per group). Statistical analysis was performed using one-way ANOVA followed by Tukey’s post hoc test. Different superscript letters indicate significant differences between groups (*p* < 0.05): ^(a)^ vs. Saline-CD; ^(b)^ vs. Saline-HSD; ^(c)^ vs. OVA-CD.

**Table 2:** mRNA expression levels in gonadal adipose tissue.

M2 Markers	OVA-CD	OVA-HSD	Level of Significance
Arginase-1	1.32 ± 0.07	0.61 ± 0.05	****
CD206	1.46 ± 0.10	0.99 ± 0.07	**
M1 Markers			
iNOS	1.44 ± 0.15	2.13 ± 0.18	*
CD11c	0.41 ± 0.09	1.87 ± 0.19	****
Pro-inflammatory Cytokines			
TNF-α	1.99 ± 0.17	4.70 ± 0.29	****
IL-6	1.30 ± 0.06	2.62 ± 0.13	****
IL-1β	2.25 ± 0.19	4.78 ± 0.22	****
Regulatory Cytokines			
IL-5	1.59 ± 0.103	0.43 ± 0.04	****
IL-10	2.84 ± 0.20	0.62 ± 0.03	****
Adipokines			
Leptin	0.67 ± 0.04	1.74 ± 0.09	****
Adiponectin	1.14 ± 0.15	0.39 ± 0.04	****

Data are presented as mean ± SEM (*n* = 6–9/group), normalized to B2M as the housekeeping gene and expressed as fold change relative to OVA-CD. Statistical analysis was performed using Student’s *t*-test. * *p* < 0.05; ** *p* < 0.01; **** *p* < 0.0001 versus OVA-CD.

## Data Availability

The datasets generated and analyzed during the current study are available from the corresponding author upon reasonable request.

## Supplementary Materials

The following supporting information can be downloaded at: https://www.mdpi.com/article/10.3390/nu18091475/s1. Table S1: Primer sequences used in this study.

## Abbreviations

The following abbreviations are used in this manuscript:

BAL	Bronchoalveolar Lavage
HSD	High-Sugar Diet
OGTT	Oral Glucose Tolerance Test
OVA	Ovalbumin
SAT	Subcutaneous Adipose Tissue
VAT	Visceral Adipose Tissue
