# Supplementary material for: A Short-Term High-Sugar Diet Induces Glucose Intolerance, Visceral Adipose Tissue Inflammation, and Exacerbates Experimental Allergic Asthma
Source: Nutrients. 2026 May 6;18(9):1475. doi: 10.3390/nu18091475 (PMC13165401; doi:10.3390/nu18091475)
Supplement: Supplementary file 1 [file nutrients-18-01475-s001.zip › nutrients-4280065-supplementary.pdf]

Supplementary Table S1. Primer sequences used in this study.

Gene	Forward Primer (5'→3')	Reverse Primer (5'→3')
B_2M	5′CCCCACTGAGACTGATACATACG-3	′5-CGATCCCAG TAGACGGTCTTG-3
Arginase	5′-GGA AGA GTG AGT GTG GTG GTG GTG G-3′	5′-CAG GAG AAA GGA CAC AGG TTG C-3′
CD2026	5′-TTC AGC TAT TGG ACG CGA GG-3′	5′-GAA TCT GAC ACC CAG CGG AA-3′
iNOS	5′-CTA GTG CAA AGC CCA AC-3′	5′-ATA CTG TGG ACG GGT CGA TG-3′
CD11c	5′-AGC AGG TGG CAT TGT GGG AC-3′	5′-ACC TCT GTT CTC CTC CTC-3′
TNF-α	5′-GAT CGG TCC CCA AAG GGA TG-3′	5′-GTG GTT TGT GAG TGT GAG GGT-3′
Il-6	5′- CGG AGA GGA GAC TTC ACA GAG-3′	5′-GGT AGC ATC CAT CAT TTC TTT G-3′
IL-1β	5′-GGC AGC TAC CTG TGT CTT TCC C-3′	5`-ATA TGG GTC CGA CGA CAC GAG-3`
IL-5	5′-TCA AAC TGT CCG TGG GGG TA-3′	5′-CTC GCC ACA CTT CTC TTTTTGG-3`
IL-10	5′-AGG CGC TGT CAT CGA TTT CTC-3′	5′-CTC TTC ACC TGC TCC ACT GC-3`
Leptin	5-TGC TGC AGA TAG CCA ATG AC-3′	5-GAG TAG AGT GAG GCT TCC AGG A-3′
Adiponectim	5'-TGG ATC TGA CGA CAC CAA AA-3'	5'-CGA ATG GGT ACA TTG GGA AC-3'
